# Controls on concentrations and clumped isotopologues of vehicle exhaust methane

**DOI:** 10.1371/journal.pone.0315304

**Published:** 2025-02-21

**Authors:** Jiayang Sun, Mojhgan A. Haghnegahdar, Julianne M. Fernandez, Cédric Magen, James Farquhar

**Affiliations:** 1 Department of Geology, University of Maryland, College Park, Maryland, United States of America; 2 Air Resources Laboratory, National Oceanic and Atmospheric Administration, College Park, Maryland, United States of America; 3 Cooperative Institute for Satellite Earth System Studies, University of Maryland, College Park, Maryland, United States of America; 4 Smithsonian Environmental Research Center, Edgewater, Maryland, United States of America; 5 Global Monitoring Laboratory, National Oceanic and Atmospheric Administration, Boulder, Colorado, United States of America; 6 Earth System Science Interdisciplinary Center, University of Maryland, College Park, Maryland, United States of America; University of Houston, UNITED STATES OF AMERICA

## Abstract

Methane emissions from vehicle exhaust, as a source of methane, are often overlooked. However, in areas with high vehicle activity, the emissions can be substantial. There is a notable lack of characterization regarding the variable concentrations and isotopic signatures of methane in vehicle exhaust. This gap in knowledge limits our understanding of the mechanisms of methane production in vehicles and the factors controlling concentration variations and isotopic fractionation, which also makes it difficult to identify and reduce methane emissions from vehicle exhaust. This study characterized the methane concentration ([CH_4_]), methane-to-ethane ratio (C_2_:C_1_), methane carbon and hydrogen isotopes (δ^13^C and δD), and methane clumped isotopologues (Δ^13^CH_3_D and Δ^12^CH_2_D_2_) of the vehicle exhaust methane endmember. [CH_4_] varied widely from below 1 ppm to more than 3000 ppm, potentially influenced by vehicle maintenance and operational phases. Ethane concentrations ([C_2_H_6_]) correlated with [CH_4_], yet C_2_:C_1_ varied significantly from 0.1% to 18.3%. The δ^13^C and δD values of exhaust methane were less negative than those of natural gas. A large portion of samples showed a positive linear relationship between [CH_4_], δ^13^C from -22‰ to -11‰, and δD values from -170‰ to -120‰, while their clumped isotopologues exhibit ~0.8‰ clumping in Δ^13^CH_3_D and ~-2.4‰ anti-clumping in Δ^12^CH_2_D_2_. A small portion of the samples exhibited distinct isotopic characteristics, with their δ13C and δD values either becoming significantly more positive or aligning closer to the composition of ambient air, while their Δ^12^CH_2_D_2_ values showed a marked increase, reaching between +25‰ to +33‰. These concentration and isotope characteristics show trends that can be explained by a combination of processes, including 1) methane formation in the engine, 2) methane combustion in the engine, 3) methane oxidation by the catalytic converter, and 4) mixing with air. The observed isotopic fractionation can be explained by thermo equilibrium and Rayleigh fractionations. These processes, elucidated through isotopic and clumped isotopologue analyses, underscore the intricate dynamics and controls of vehicular methane emissions.

## Introduction

In addition to carbon dioxide (CO_2_), vehicles emit other greenhouse gases (GHGs) such as methane (CH_4_) and ethane (C_2_H_6_). CH_4_ is a potent GHG and accounts for approximately 1/3 of added radiative forcing on climate with its global warming potential 80 times that of CO_2_ over a 20-year period [1]. However, these short chain alkanes emissions from vehicle exhausts are not subject to regulation or routinely monitored. Studies on vehicle exhaust have focused more on pollutants like NO_x_, SO_2_, CO, and particulate matters [2–5], with less attention given to methane and ethane [6–8]. Engine combustion models indicated that methane could be produced through pyrolysis of fuel and radical reactions, under a high-temperature engine environment [9, 10], although combustion with oxygen and subsequent catalytic oxidation at the surface of the catalytic converter should consume methane [11]. Methane emissions from vehicle exhaust were often considered negligible and were rarely included in global methane inventories [12]. Previous studies estimated global automobile methane emissions between 0.45 ± 0.12 Tg CH_4_ yr^-1^ [7] and 0.5–3.4 Tg CH_4_ yr^-1^ [13], representing about 0.06% to 0.6% of estimated global total methane emissions [12]. However, studies have highlighted that in certain urban areas, vehicular methane emissions can significantly impact the local methane budget. For instance, isotope data suggested that automobile exhaust contributed up to 30% of the near-surface air methane in Nagoya, Japan, in 1993 [14]. Two traffic air samples from the LAX parking garage and Sepulveda tunnel in Los Angeles, USA, displayed isotopic signals similar to those of vehicle exhaust methane but only had background methane concentrations ([CH_4_]), suggesting the sampled areas were saturated with vehicle exhaust [15].

Better constraining vehicular methane emissions is necessary for supplementing the methane budget, especially in urban areas, which have been identified as methane emission hotspots [16–19]. Urban areas have stationary high-flux point sources like landfills [20, 21], wastewater treatment plants [22], and natural gas infrastructure [23], and also prevalent mobile low-flux sources like vehicle exhaust. While an individual vehicle may contribute minimally, the collective effect of numerous vehicles can slightly elevate urban background [CH_4_]. However, the low enhancement level makes it challenging to identify and quantify the contributions from vehicle sources, if proxies other than [CH_4_] were not used.

Methane carbon and hydrogen isotopes (δ^13^C and δD) and ethane-to-methane ratio (C_2_:C_1_) are frequently used for apportionment of major methane sources such as natural gas leaks, microbial emissions, and agricultural contributions [15, 24–26]. These indicators are particularly effective in differentiating between methane of microbial and thermogenic origins, as these sources usually yield methane with distinct isotopic signatures [25, 27]. Thermogenic methane, which is formed under high pressure and temperature and is often found in natural gas reserves, generally exhibits less negative δ^13^C and δD values and higher C_2_:C_1_ [28, 29]. In contrast, microbial methane, such as those from wetlands, presents more negative δ^13^C and δD [25], coupled with lower (if not zero) C_2_:C_1_. Although vehicle exhaust methane originates from fossil fuels, it cannot be simply classified as thermogenic methane because the formation and subsequent alteration processes (pyrolysis, combustion, and catalytic oxidation) can strongly fractionate methane isotopes.

Recent advances in measurement techniques have enabled the measurement of the relative abundance of methane doubly-substituted isotopologues (i.e. ^13^CH_3_D and ^12^CH_2_D_2_, also referred to methane clumped isotopologues) [30–34]. The capability of distinguishing methane from various sources using methane clumped isotopologues have been preliminarily explored [35]. Thermogenic methane usually exhibits thermodynamic equilibrium clumping signals [35–38], whereas microbial methane from surface and lab environments may show strong anti-clumping signals [35, 39, 40]. Oxidation processes, including microbial aerobic oxidation [41, 42], microbial anaerobic oxidation [42, 43], and atmospheric sink reactions with OH and Cl radicals [34, 44–46], tend to cause stronger clumping signals in residual methane.

Existing data is sparse in defining the isotopic composition of the vehicular methane endmember [14]. The clumped isotopologue signals of vehicle exhaust methane have not been measured before. This limits our ability to identify the contributions of vehicle exhaust and refine global methane isotope budgets [34, 47, 48]. It also restricts our understanding of methane-related reactions that happened in the engine and catalytic converter environments, where key isotopic fractionation parameters of pyrolysis and catalytic oxidation could be obtained. These parameters are crucial for understanding methane geochemical processes such as thermogenic methane formation [49], microbial methane oxidation [42, 43, 50], and atmospheric sink reactions [34, 44–46].

This study will fill this data gap by characterizing the isotopic (δ^13^C and δD) and clumped isotopologue (Δ^13^CH_3_D and Δ^12^CH_2_D_2_) compositions of vehicle exhaust methane, while also collecting [CH_4_] and ethane concentration ([C_2_H_6_]) information. The dataset enables us to define the isotopic signatures of the vehicular methane endmember, determine if clumped isotopologues can be used to distinguish vehicular methane from other methane emissions, and explore processes and controlling factors in vehicular methane production and isotopic fractionation.

## Materials and methods

### Sampling strategies and procedures

Although methane emissions from vehicle exhaust are not regulated, studies on other vehicle exhaust pollutants like CO and NO_x_ suggested that emission profiles can vary substantially among vehicles [51], which could also apply to methane. The variation is expected to be influenced by factors such as vehicle type, engine design, ignition method, fuel type, air-to-fuel ratio, catalytic converter conditions, and operating conditions [51]. To pinpoint endmember signatures, our study involved collecting exhaust samples from a wide array of vehicles, which included different brands, years of manufacturing, engine configurations, and fuel types.

Two sampling and measurement campaigns were carried out and summarized in Table 1. The first campaign aimed to delineate a range of isotope and isotopologue signatures for vehicle exhaust methane. We intentionally collected exhaust gases from some old and poorly maintained vehicles, with the assumption that these vehicles have different exhaust methane compositions compared to newer vehicles. The second campaign did not involve gas collection for isotope measurements but focused on expanding the [CH_4_] and [C_2_H_6_] profiles. We employed a Mira Ultra LDS Infrared multipass laser gas analyzer (Aeris#302) from Aeris Technologies Inc. for direct, real-time exhaust monitoring. Volunteer vehicles on the University of Maryland (UMD) campus were randomly sampled, predominantly consisting of common household vehicles aged between 0 to 15 years.

**Table 1:** Compiled information on sampled vehicles. Samples collected or concentrations measured while the vehicles were idling, revving, after revving, right after a cold start, or after warmed up, were labeled as ‘idle’, ‘accelerate’, ‘post-accelerate’, ‘cold’, or ‘hot’, respectively. Detailed definitions of these vehicle conditions and operational phases can be found in Supporting Information S2 Note in S1 File.

**Validation test sampling**	**Model Year**	**Brand**	**Model**	**CH**_**4**_ **(ppm) idle**	**C**_**2**_**H**_**6**_ **(ppb) idle**					**Collected?**	**Notes**
	1970	Dodge	Charger	299.3	20785.4^a^					Yes, Idle	No catalyst
	2017	Chevrolet	Sonic	9.8	24.4					Yes, Idle	/
**First sampling campaign**	**Model Year**	**Brand**	**Model**	**CH**_**4**_ **(ppm) cold**		**C**_**2**_**H**_**6**_ **(ppb) cold**	**CH**_**4**_ **(ppm) hot**		**C**_**2**_**H**_**6**_ **(ppb) hot**	**Collected?**	**Notes**
	1970	Dodge	Charger	1183.5		41412.5^a^	443.0			Yes, hot	No catalyst
	1981	BMW	528i	3101.8	1924.0		667.5	230.0		Yes, hot*2&cold*2	Poor condition
	2013	Kohler	XT675 (Mower)				204.0			Yes, hot	No catalyst
	1999	Volvo	S70	195.1			112.5			Yes, hot&cold	/
	1995	Mazda	MX-5 Miata				126.4			Yes, hot	/
	/	Ford	F-250				61.2			No	Long idling
	2017	Chevrolet	Sonic				2.0			Yes, hot	/
	2005	Gillig	Transit Bus				2.0			Yes, hot	Diesel
	2013	Gillig	Transit Bus				1.4			Yes, hot	Diesel
	2006	Ford	F-750 (Trash Truck)				9.2			Yes, hot	Diesel
**Second sampling campaign**	**Model Year**	**Brand**	**Model**	**CH**_**4**_ **(ppm) idle**	**C**_**2**_**H**_**6**_ **(ppb) idle**	**CH**_**4**_ **(ppm) accelerate**	**C**_**2**_**H**_**6**_ **(ppb) accelerate**	**CH**_**4**_ **(ppm) post-accelerate**	**C**_**2**_**H**_**6**_ **(ppb) post-accelerate**	**Collected?**	**Notes**
	2015	Honda	Civic EX	5.2^a^	6.3	36.5^a^	697.9^a^	/	/	No	/
	2023	Chevrolet	Silverado	0.1	2.6	/	/	/	/		Brand new
	2017	Toyota	Prius Prime Hybrid	2.1	4.0	/	/	/	/		On electric engine
	2011	Hyundai	Elantra	15.7^a^	188.23^a^	77.36^a^	1183^a^	21.5^a^	125.5^a^		/
	2016	Toyota	Camry	35.9^a^	500.1^a^	154.8^a^	12973.5^a^	73.7^a^	7347.2^a^		/
	2013	Nissan	Altima	28.4^a^	1665.8^a^	217.6^a^	23739^a^	2.3	72.69^a^		Wet exhaust
	2019	Audi	A5	0.6	4.9	44.7	700.32^a^	7.1^a^	40.5		/
	2012	Honda	Civic coupe	20.3^a^	1271^a^	72.3^a^	5725.3^a^	1.0	4.8		/
	2009	Honda	Accord	58.1^a^	6816^a^	116.4^a^	20266.1^a^	4.03^a^	14.4		V6
	2015	Nissan	Altima	24.5^a^	148.73^a^	18.2	107.3^a^	9.3^a^	26.9		/
	2017	Alfa Romeo	Giulia Q4	25^a^	1460.9^a^	19.2	847.3^a^	10^a^	219.3^a^		Turbo
	2019	Hyundai	Kona	1.4	3.0	24.5	794.5^a^	0.1	3.0		/
	2023	Lexus	GX460	0.3	3.4	5.7	6.3	0.0	3.2		V8, brand new
	2018	Toyota	RAV4	180.2^a^	32815.7^a^	0.4	19.5	51.2^a^	4150.2^a^		/

^a^Concentrations were extrapolated with the assumption that the Aeris responded linearly to the concentration. These concentrations with label ^a^ could be considered as semi-quantitative. Concentrations without labels are strictly quantitative. See section “Methane and ethane concentration measurements and calibrations for more details.

**Fig 1:**
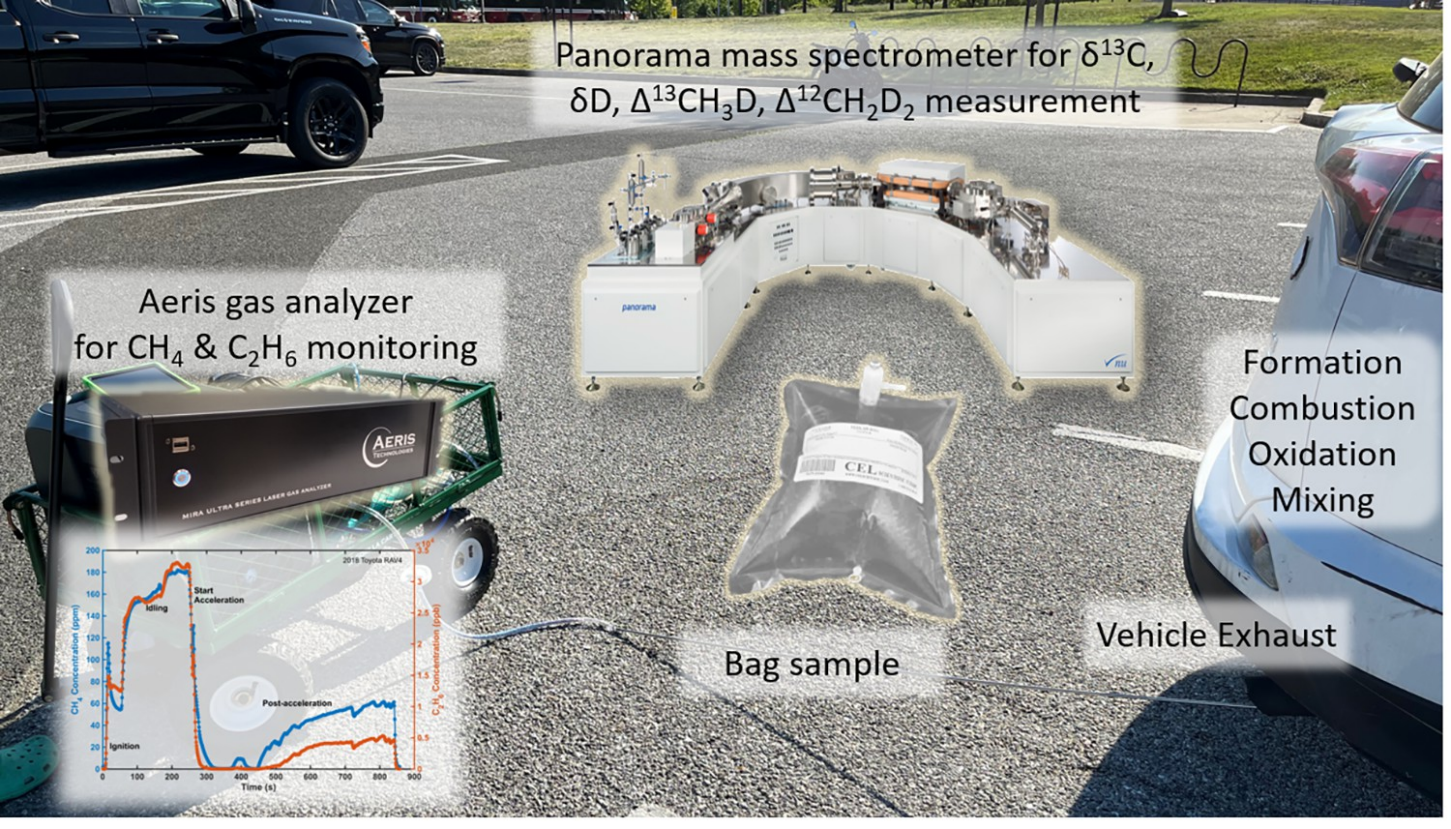
Schematic diagram of sampling and measurement processes.

Fig 1 schematically illustrates the sampling and measurement settings. The large-volume sampling method and real-time monitoring procedures are described in Supporting Information S1 Note in S1 File. Descriptions of each sampled vehicle can be found in Supporting Information S2 Note in S1 File. In the subsequent text, we refer to each sampled vehicle and the corresponding sample using the format”year make model”, followed by a brief description of the state (“cold” or “hot”), sometimes an operation status (ignition, idling, revving, or post-acceleration), and sometimes a numerical suffix (1 or 2) when necessary. For example, “1981 BMW 528i cold2” refers to the second sample collected from the 1981 BMW 528i right after a cold start in the idling state). In the figures, the “year make” information is omitted for clarity. Definitions of these vehicle states can be found in Supporting Information S2 Note in S1 File.

### Methane and ethane concentration measurements and calibrations

Most of the methane concentration data, as well as all ethane concentration data, were acquired using the Aeris MIRA Ultra analyzers (#302). The Aeris employs mid-Infrared laser absorption spectroscopy, detecting the characteristic transition frequencies of target molecules. According to our tests, this Aeris #302 has a typical precision (1SD) of 1.6 ppb for CH_4_ and 0.16 ppb for C_2_H_6_ with a one-second integration time.

The Aeris instrument was calibrated at the NOAA Air Resources Lab, College Park, Maryland. We used Aeris to measure three tank compressed standard gases with known methane concentrations (#19 at 1925.19 ppb, #14 at 2204.10 ppb, and #18 at 2528.95 ppb) and zero air for methane calibration. For ethane calibration, we used a mass flow controller to mix 2 standard litre per minute zero air with compressed house natural gas (known 4.83 ppm ethane) from 0 to 20 standard cubic centimeters per minute at 2 standard cubic centimeters per minute increments, to serve as standard gases. Each standard sample was measured for over 5 minutes to ensure thorough flushing and eliminate instrument delay. We used the average of the last 3 minutes of each measurement period to calculate the average. The measured averages were linearly fitted against theoretical concentrations to obtain a correction equation that would be applied to real measurements. The instrument uncertainty was derived from the 1SD of the last 3 minutes of zero air measurements. However, these calibration procedures are designed for ambient air monitoring, but not for direct vehicle exhaust measurements, which often exceed calibration ranges. For the data measured by Aeris, only methane concentrations between 0–2.53 ppm and ethane concentrations between 0–51.3 ppb can be considered strictly quantitative. Data beyond this calibration range are extrapolated based on the assumption of the instrument’s linear response to concentrations, with the upper limits of this linear response typically being 10,000 ppm for CH_4_ and 1,000 ppm for C_2_H_6_, according to the manufacturer’s specifications. Direct measurements of vehicle exhaust using Aeris are discouraged due to potential optical contamination and the possibility that concentrations may exceed the instrument’s linear response range. In this study, no concentration values exceeded the manufacturer’s reported upper limits for linear response. Thus, concentration measurements in this study are generally reliable. If the assumption of linear response is deemed invalid, then methane concentration data above 2.53 ppm and ethane concentration data exceeding 51.3 ppb measured by Aeris should be considered semi-quantitative.

For bag samples collected before we obtained the Aeris, methane concentrations were determined using a Shimadzu^®^ GC-8AIF Gas Chromatograph (GC). Ethane concentrations were not measured. Injected from a 1 ml sample loop, samples were carried by N_2_ carrier gas at approximately 60 ml/min through a stainless-steel GC column with an inner diameter (ID) of 1/8 inch, packed with 2.5 m of 80/100 pre-conditioned HayeSep Q. H_2_, O_2_, N_2_, and CO were eluted, while CH_4_ and CO_2_ were retained in the column. Subsequently, N_2_ carrier gas again carried residues in the column through another stainless-steel column with an ID of 1/8 inch, packed with 0.5 m of 100/100 Shimalite Q. The columns’ temperatures were held at 80°C. CH_4_ peak signals were detected using a Flame Ionization Detector (FID), with air and H_2_ gas flows set to approximately 300 ml/min and 50 ml/min, respectively, for combustion in the FID. Concentrations measured by GC are all quantitative.

### Extraction and purification of bag samples

Before taking measurements, each bag sample underwent extraction and purification processes to isolate pure methane from other gases, following the procedures outlined in [34]. Briefly speaking, the sample gas was dried using silica gel and directed through a large U-trap filled with Hayesep DB, which was cooled with liquid nitrogen. This step effectively trapped almost all sample gas in the large U-trap except partially removing N_2_ and O_2._ Upon thawing the large U-trap, the trapped gas was redirected through a small U-trap filled with Hayesep DB, also cooled with liquid nitrogen. At this stage, the majority of N_2_ and O_2_ were removed, while CH_4_ and Kr were fully retained. The trapped gas was warmed to -120°C using an ethanol slush and held at this temperature for 30 minutes to further remove N_2_ and O_2_. The residual gas underwent GC separation for final purification. The GC column was packed with 25 feet of 60–80 mesh MolSieve 5A and maintained at 56°C.

Compared to the method for normal air samples [34], the extraction of vehicle exhaust samples requires two additional steps to remove substantial amounts of CO_2_ and H_2_O. Failure to remove CO_2_ and H_2_O could result in process inefficiency and possibly cause sample fractionation. The sample gas was first reacted with a high-concentration (~50%w/v, ~20°C) NaOH aqueous solution by pulling the gas through a gas frit into the solution. The NaOH solution was aerated with N_2_ for 3 hours before use, to remove CH_4_ that has been reported in NaOH pellets [52]. A glass condenser cooled to liquid nitrogen temperature was utilized subsequently to trap any remaining CO_2_ and H_2_O. Pressure monitoring on the purification line confirmed the efficiency of these added steps in removing CO_2_ and H_2_O. The vehicle exhaust samples also contained other impurities, which were removed during the bubbling process, as evidenced by the NaOH solution turning yellow and forming an oily layer. Given the substantial restriction on the gas flow rate by the gas frit and NaOH solution, the extraction process could take more than 8 hours, instead of 5 hours for normal air samples. Hence, we rigorously inspected and reinforced the extraction line prior to use for optimal airtightness.

### Methane isotopes and isotopologues measurement

The pure methane was analyzed using the Panorama mass spectrometer for isotope and isotopologue measurements. The Panorama is a high-resolution, high-sensitivity, double-focusing gas-source mass spectrometer with mass resolving power (MRP) that can exceed 50,000. This high MRP allows Panorama to distinguish between isotopologue ion beams with closely aligned masses such as ^13^CH_3_D^+^, ^13^CH_5_^+^, and ^12^CH_2_D_2_^+^. Detailed specifications of the Panorama instrument are provided by [32]. The methodologies and configurations used for methane clumped isotopologue measurements in the UMD lab are detailed in [34].

Bulk carbon and hydrogen isotopes are reported in ratio differences (δ notation):

δ13Csample−standard(‰)=((C13C12)sample(C13C12)standard−1)*1000

δDsample−standard(‰)=((DH)sample(DH)standard−1)*1000

Where the standards are usually Vienna Pee Dee Belemnite (V-PDB) for carbon isotopes and Vienna Standard Mean Ocean Water (V-SMOW) for hydrogen isotopes [53, 54].

Methane clumped isotopologue notations can be defined in a similar manner to carbon and hydrogen isotopes:

δ13CH3D(‰)=((C13H3DC12H4)sample(C13H3DC12H4)standard−1)*1000

δ12CH2D2(‰)=((C12H2D2C12H4)sample(C12H2D2C12H4)standard−1)*1000

Where the standard could be methane characterized by V-PDB carbon isotope composition and V-SMOW hydrogen isotope composition, and followed intra-molecular stochastic distribution, which refers to the state of purely random combinations of ^12^C, ^13^C, H, and D intramolecularly to form methane molecules [32], i.e.:

$$X_{13_{CH_{3}D,\;stochastic}}=4*X_{13_{C}}*X_{D}*{\left(X_{H}\right)}^{3}$$

$$X_{12_{CH_{2}D_{2},\;stochastic}}=6*X_{12_{C}}*{\left(X_{D}\right)}^{2}*{\left(X_{H}\right)}^{2}$$

$X_{13_{C}}$ and *X*_D_ represent the proportions of ^13^C and ^2^H atoms in the total carbon and hydrogen atoms, respectively. The coefficients 4 and 6 are derived from the symmetry of the isotopically-substituted methane molecules.

A more commonly adopted notation for methane clumped isotopologue describes the level of enrichment or depletion of ^13^CH_3_D and ^12^CH_2_D_2_ relative to the stochastic distribution within the sample [32, 55]:

$$\Delta^{13}CH_{3}D(‰)=(\frac{X_{13_{CH_{3}D}}}{X_{13_{CH_{3}D,\;stochastic}}}-1)*1000$$

$$\Delta^{12}CH_{2}D_{2}(‰)=(\frac{X_{12_{CH_{2}D_{2}}}}{X_{12_{CH_{2}D_{2},\;stochastic}}}-1)*1000$$

$X_{13_{CH_{3}D}}$ and $X_{12_{CH_{2}D_{2}}}$ are the proportion of ^13^CH_3_D and ^12^CH_2_D_2_ molecules in the total methane molecules of the sample. Under these definitions, the clumped isotopologue cap-delta signals of methane are independent from the sample’s bulk ^13^C and D abundances. In practical applications, we use approximate formulas to calculate Δ^13^CH_3_D and Δ^12^CH_2_D_2_ [34]:

Δ13CH3D(‰)=((1+δ13CH3D1000)(1+δ13CH41000)*(1+δ12CH3D1000)−1)*1000

Δ12CH2D2(‰)=((1+δ12CH2D21000)(1+δ12CH3D1000)2−1)*1000

The errors introduced by using these approximations are much smaller than the measurement uncertainties.

## Results and discussion

### Methane concentration of vehicle exhaust

**Fig 2:**
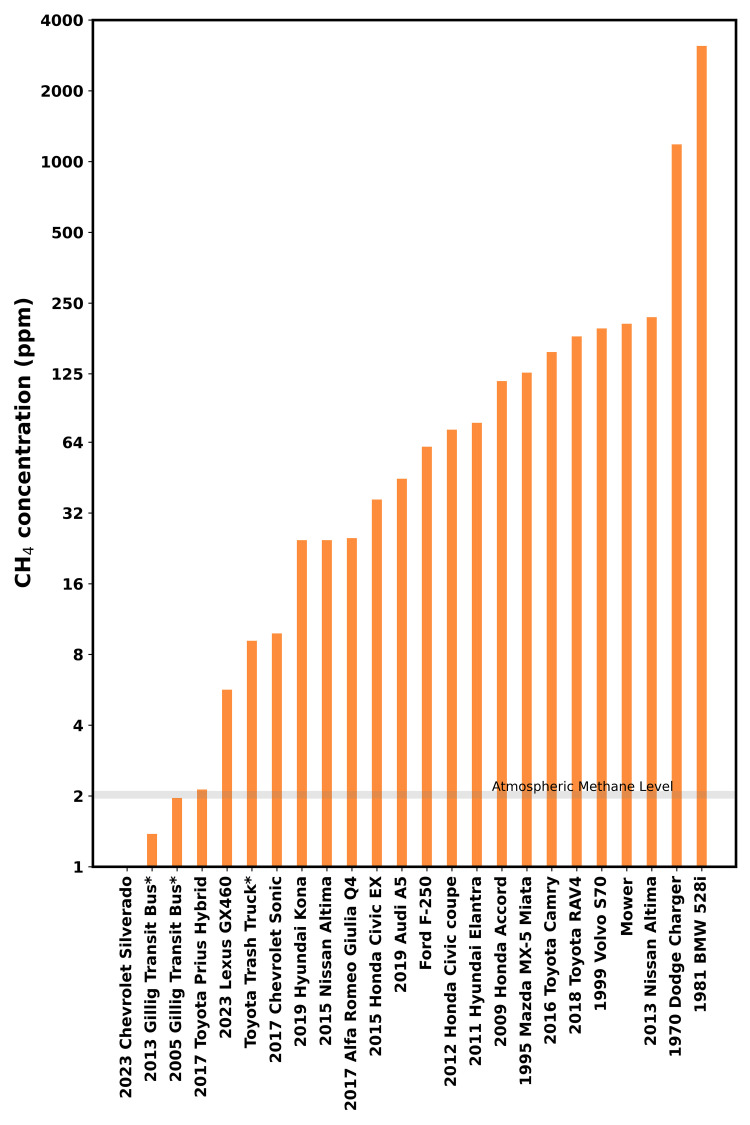
Methane concentrations of vehicle exhaust samples. The plotted concentrations are peak values among the vehicle operational cycle or states; therefore, the heights of the bars are not fully representative of the total emission fluxes from the corresponding vehicles. Asterisks (*) in labels mark diesel vehicles. The Y-axis is scaled logarithmically. Detailed information on individual vehicles can be found in Section “Sampling strategies and procedures”, Table 1, and Supporting Information S2 Note in S1 File.

Our dataset reveals that vehicular exhaust contains significant amounts of methane, contrary to the conventional assumption that high-temperature combustion in engines should oxidize nearly all methane. Methane concentrations are reported in Fig 2 and Table 1, with the highest exceeding atmospheric levels by a thousand times and the lowest below atmospheric levels. More discussions for each vehicle can be found in Supporting Information S3 Note in S1 File. While lacking quantitative parameters to assess vehicle condition, we infer that well-maintained and newer vehicles tend to have lower [CH_4_], whereas vehicles in suboptimal conditions (for instance, right after cold start, with loose plug wires, or with malfunctioning catalytic converters) exhibit elevated [CH_4_] in the exhaust.

Continuous monitoring of vehicle exhaust throughout the operation cycles (ignition-idling-revving-idling) revealed two key observations: 1) vehicles usually have transient [CH_4_] spikes at the moment of ignition and the onset of revving (S2 Fig in S1 File); 2) revving may result in either a moderate increase (S2b Fig in S1 File) or a substantial decrease (S2c Fig in S1 File) in [CH_4_]. This behavior may reflect combustion dynamics within the engine influenced by fuel delivery systems. As extra fuel is added for acceleration, contemporary vehicles employ computerized control of fuel injection and ignition timings, optimizing air-to-fuel ratio and combustion efficiency [56], and may have smaller enhancements compared to older engines with carburetors.

Overall, [CH_4_] data imply that vehicular exhaust emissions may follow a fat-tail distribution (i.e., a minority of events/units contribute most of the emissions), a pattern similar to that of natural gas system emissions [57]. Even if vehicles in poor condition comprise only a small fraction of the total fleet, they could still account for a significant proportion of vehicular methane emissions.

### Ethane concentration of vehicle exhaust

A wide range of [C_2_H_6_] was observed in vehicle exhaust (S3 Fig in S1 File), extending from a few ppb to more than 30,000 ppb. As can be seen from S2 Fig in S1 File, [CH_4_] and [C_2_H_6_] in vehicle exhaust increased and decreased almost simultaneously, indicating that they were generated and consumed by the same processes in vehicle engines and catalytic converters.

**Fig 3:**
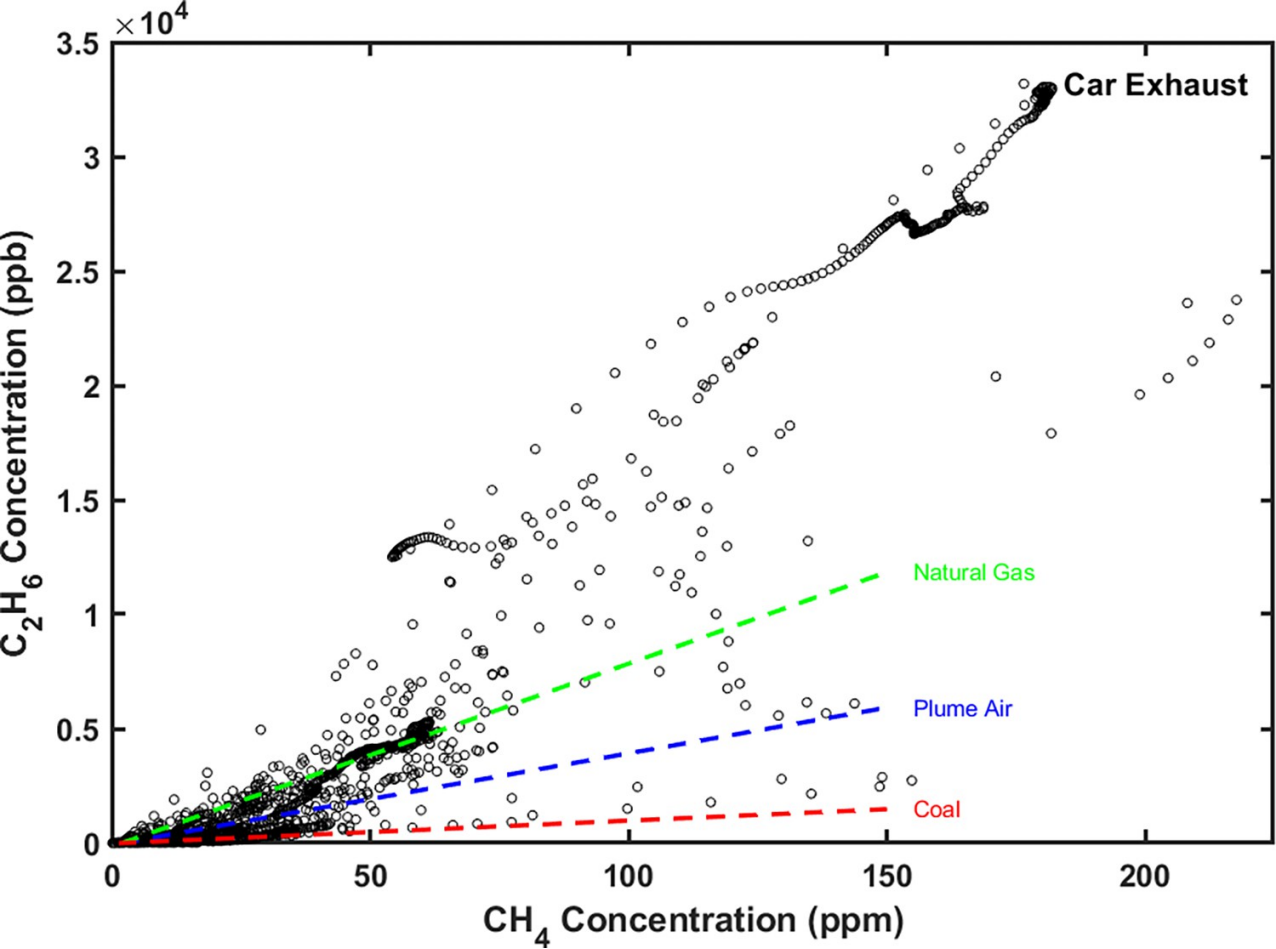
[CH_4_] of all sampled vehicle exhausts in the second sampling campaign. **[C**_**2**_**H**_**6**_**] vs.** The campaign continuously monitored [CH_4_] and [C_2_H_6_] of the exhaust during operation cycles (ignition, idling, acceleration, post-acceleration). Three reference lines indicate approximate C_2_:C_1_ for Baltimore natural gas (green), plume air observed in the air over the Baltimore-Washington Area (blue), and coal emissions (red).

Despite the concentrations changing simultaneously, the [C_2_H_6_]-[CH_4_] plot (Fig 3) reveals substantial variability in C_2_:C_1_, ranging from approximately 0.1% to 18.3% across different vehicles. Although the production mechanisms for CH_4_ and C_2_H_6_ in vehicles are linked, preferences for producing CH_4_ or C_2_H_6_ differ based on the vehicle’s condition and operational state. Larger ratios often correlated with higher [C_2_H_6_], suggesting that vehicles in poorer conditions that produced more CH_4_ also contributed higher amounts of C_2_H_6_. Very high [CH_4_] and [C_2_H_6_] in some vehicles could yield spikes during roadside and/or on-road monitoring.

Aircraft monitoring studies reported C_2_:C_1_ of 3.3% ± 0.35% and 4.31% ± 0.63% in the air over the Baltimore-Washington Area during the winters of 2015 and 2016, respectively [19]. They also reported data from natural gas samples provided by the Baltimore Gas & Electric Company, showing C_2_:C_1_ of 8.40% and 7.81% for the same periods. A ratio of 2.3% has been reported in the air above the Marcellus Shale production region [18], with a ratio of 6.7% from direct Marcellus Shale well samples [58]. Stable coal vents in the San Juan Basin were reported to have C_2_:C_1_ of 1.28% ± 0.11% [59]. In general, coal emissions have a lower C_2_:C_1_ than natural gas, often less than 1 wt-% [60]. The highly variable C_2_:C_1_ in vehicle exhaust overlapped the range from coal piles to natural gas wells, thus the ratio alone may not serve as an indicator for differentiating vehicle exhaust from thermogenic sources, but the variability in C_2_:C_1_ as vehicles transitioned through different operational states on the road might provide a signature for use during monitoring that distinguishes vehicle emissions from more homogenous sources like natural gas and coal.

### Carbon and hydrogen isotopes of vehicle exhaust

**Fig 4:**
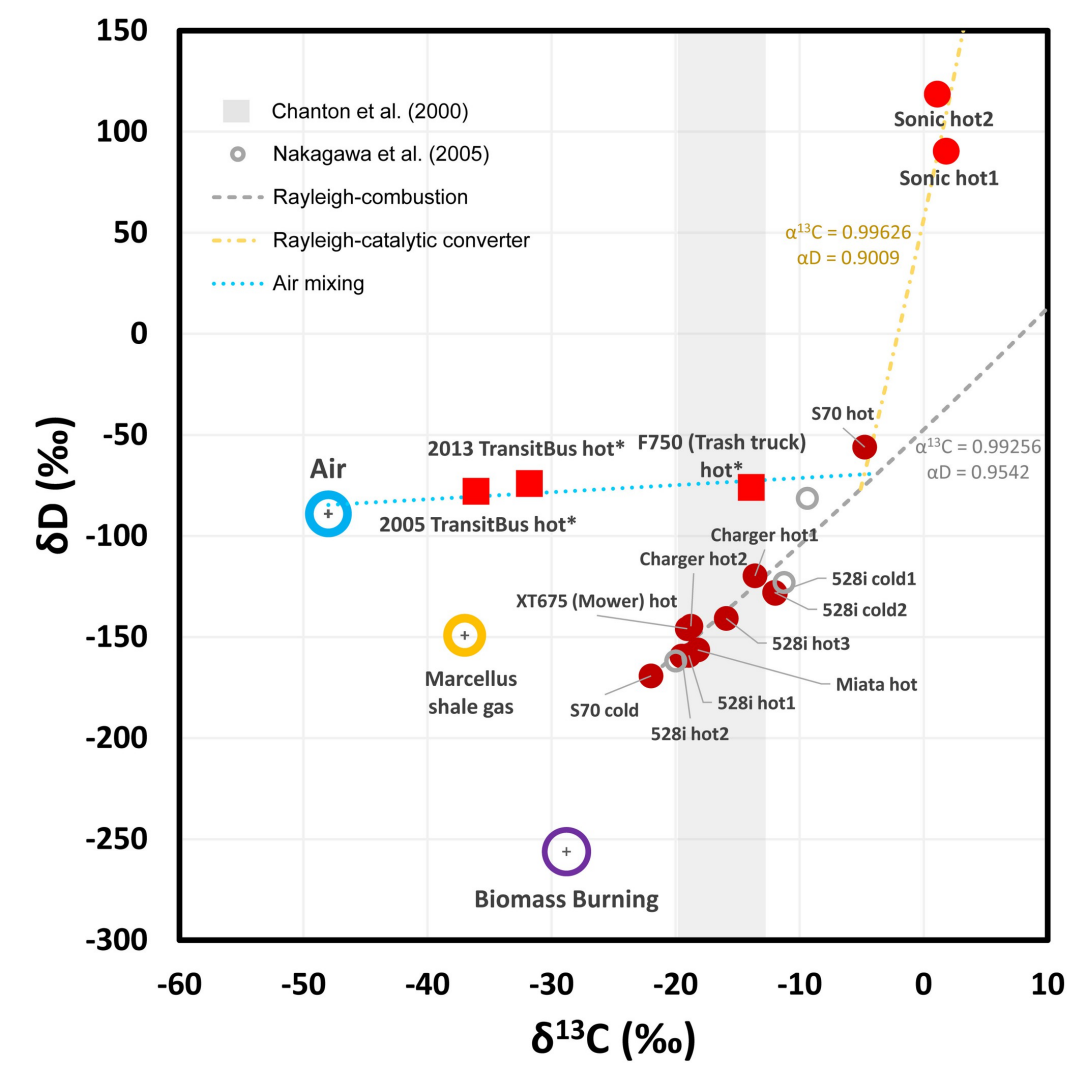
δD-δ^13^C plot of vehicle exhausts. Red solid circles are for gasoline vehicles. The red squares are for diesel vehicles. Approximate compositions of air (blue ring), natural gas (yellow ring), and biomass burning (purple ring) methane are shown in the figure. Three averaged exhaust compositions derived from [14] are shown as gray circles. The gray shaded area marks the estimated range of exhaust carbon isotopes reported by [13]. Error bars (2SD) with these measured data points are smaller than the symbols. The gray dashed line and the yellow dash-dotted line are isotopic compositions of the residue methane following the Rayleigh fractionation caused by the combustion and catalytic converters, from different initial compositions, with different isotopic fractionation factors. The blue dotted line is the mixing line of air with an exhaust.

The δ^13^C and δD of vehicle exhaust exhibit considerable variability (Fig 4 and Table 2). Most vehicle exhaust methane samples are more enriched in ^13^C than natural gas methane and biomass burning methane, and more enriched in deuterium (D) relative to biomass burning methane but similar to natural gas methane. In comparison to the δ^13^C of gasoline, which has been reported to range from -34‰ to -21‰ with an average of approximately -27‰ [61], methane emissions from vehicle exhaust present different levels of enrichment in ^13^C (δ^13^C > -22‰ for all vehicle samples, with the highest δ^13^C reaching 2‰) but show no significant difference in δD values compared to the range reported for a global set of gasoline, which varies between -70% and -145‰ [62].

**Table 2:** Methane concentrations, carbon isotopes, hydrogen isotopes, and clumped isotopologues of vehicle exhaust samples collected in the first sampling campaign. Uncertainties reported in this table are 2SD.

	[CH_4_] ppm	δ^13^C ‰	σδ^13^C ‰	δ^13^CH_3_D ‰	σδ^13^CH_3_D ‰	δD ‰	σδD ‰	δ^12^CH_2_D_2_ ‰	σδ^12^CH_2_D_2_ ‰	Δ^13^CH_3_D ‰	σΔ^13^CH_3_D ‰	Δ^12^CH_2_D_2_ ‰	σΔ^12^CH_2_D_2_ ‰
**S70 hot**	112.49	-4.76	0.15	-60.00	1.30	-56.06	0.49	-113.41	4.84	0.6	1.5	-5.0	5.5
**S70 cold**	195.09	-21.99	0.03	-186.14	0.72	-169.19	0.20	-310.99	1.81	1.6	0.9	-1.8	2.7
**528i cold1**	3101.78	-11.97	0.03	-138.62	0.67	-128.33	0.06	-239.96	0.97	0.2	0.8	0.3	1.3
**528i cold2**	1923.95	-12.02	0.03	-137.75	0.64	-127.90	0.22	-238.14	1.96	0.7	0.8	1.7	2.6
**528i hot1**	667.46	-18.93	0.02	-175.35	0.42	-159.06	0.09	-294.62	1.27	-0.4	0.5	-2.5	1.8
**528i hot2**	667.46	-19.51	0.05	-173.43	0.90	-159.44	0.62	-294.70	3.22	2.9	1.3	-1.8	4.8
**528i hot3**	230.00	-15.91	0.01	-154.26	0.29	-140.82	0.09	-265.26	1.45	0.3	0.4	-4.7	2.0
**Charger hot1**	/	-13.61	0.02	-130.91	0.82	-119.73	0.09	-224.32	1.40	0.9	1.0	1.0	1.8
**Charger hot2**	443.03	-18.76	0.46	-159.99	0.75	-144.58	1.56	-271.23	2.30	0.8	2.1	-4.1	4.8
**Miata hot**	126.39	-18.21	0.03	-170.99	0.62	-156.40	1.55	-290.94	1.81	0.9	2.0	-3.7	4.5
**Sonic hot1**	1.38	1.81	0.11	95.61	2.10	90.39	0.77	218.78	6.55	3.0	2.1	25.1	5.7
**Sonic hot2**	1.96	1.10	0.02	120.96	0.65	118.64	2.14	285.99	4.44	1.0	2.0	27.7	5.3
**XT675 (Mower) hot**	204.01	-19.07	0.05	-161.69	0.82	-145.91	0.26	-274.84	2.21	0.6	1.0	-5.9	3.1
2005 TransitBus hot*	1.96	-36.11	0.04	-110.05	0.15	-77.83	0.15	-123.96	1.98	1.2	0.2	30.1	2.4
2013 TransitBus hot*	1.38	-31.79	0.05	-101.85	1.00	-74.04	0.43	-114.39	3.15	1.8	1.2	32.9	3.8
F750 (Trash truck) hot*	9.15	-13.91	0.01	-87.93	0.28	-75.93	0.09	-138.32	1.33	0.9	0.3	9.1	1.6

**Fig 5:**
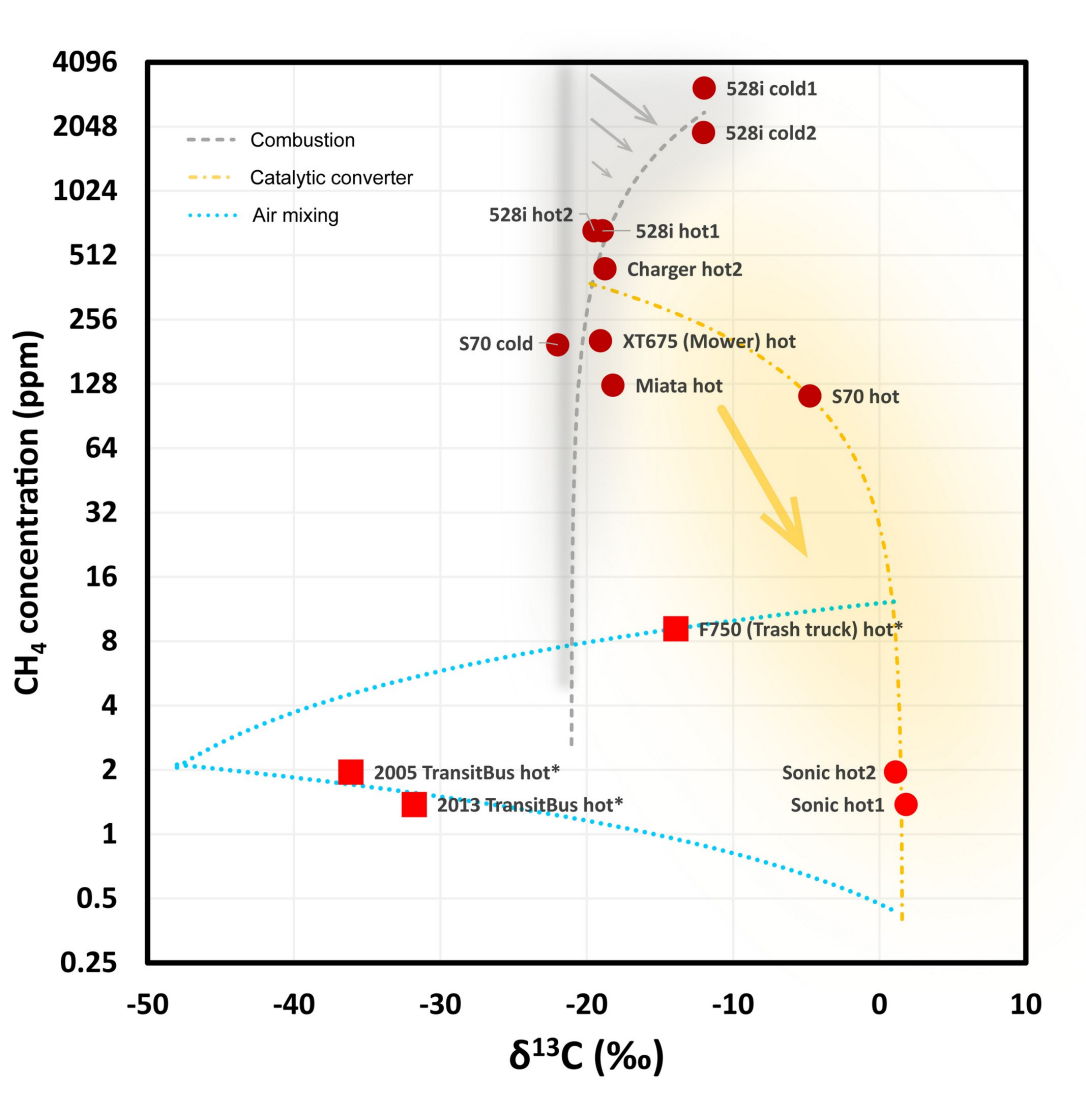
[CH_4_]-δ^13^C plot of vehicle exhausts. The [CH_4_] axis is on the log scale. Symbols, colors, and error bars are the same as in Fig 4. The two blue dotted curves are linear mixing arrays for the trash truck, and two school shuttle buses, with air respectively. The gray dashed line is plotted by linear fittings of the majority of vehicles, and the yellow dash-dotted line is the linear fitting of samples from 2017 Chevrolet Sonic hot. The gray band indicates the methane produced during the high-temperature methane formation with consistent δ^13^C but different [CH_4_]. The formed methane underwent fractionation in the direction indicated by the gray arrow through combustion with α_13C_ = 0.9926, resulting in compositions as suggested by the gray area. The yellow arrow shows the effect of methane oxidation on the catalytic converter with α_13C_ = 0.9985, resulting in methane compositions represented by the yellow area. The gray and yellow areas are schematic ranges only.

A positive linear correlation between δ^13^C and δD values is observed in the majority of exhaust samples (Fig 4). This correlation aligns with a previous work [14], which attributed this relationship to the effect of catalytic converters. A similar trend was observed with δ^13^C values and [CH_4_] [13], where higher [CH_4_] correlated with more negative δ^13^C values, suggesting this pattern relates to combustion efficiency, which is also shown in our data (Fig 5). Exhaust samples from a few cleaner gasoline vehicles and all diesel vehicles proved to be exceptions from linear relationships, indicating that methane emitted from vehicle exhaust is the result of multiple processes that produce and consume it.

We considered four processes to explain the observed trends and outliers, including (1) the formation of methane under high-temperature burning (Process #1 methane formation), followed by (2) the combustion of methane while still under high-temperature before expelled from the cylinder to the exhaust stroke (Process #2 methane combustion), (3) the oxidation of methane in the exhaust stroke by the catalytic converter (Process #3 catalytic oxidation), and (4) mixing of exhaust methane with air methane (Process #4 air mixing).

The formation of methane occurs primarily through collisions of methyl radicals with hydrogen radicals, following radical chain reactions starting from gasoline [9, 10]. Though the reaction $CH_{3}∙+H\cdot +M↔CH_{4}+M$ thermodynamically favors the formation of methane, methane acts as a catalyst in the decomposition of small carbon-hydrogen-oxygen compounds such as H·, ·OH, HO2·, HCO·, CO·, and CH3O·[10], causing newly formed methane to approach inter- and intramolecular isotope equilibrium, within such a high temperature closed system. There are no reference values available for the fractionation coefficient between gasoline and formed methane resulting from this step. If we assume the δ^13^C of gasoline is -27‰ [61], then the fractionation for this step may be +4–5‰. If assuming minimal fractionation [13], then local gasoline δ^13^C composition is approximately -21‰.

In the later parts of the combustion process, the production of alkyl radicals slows due to the consumption of fuel, and the consumption of methane starts by combustion with O_2_. The combustion process preferentially consumes ^12^CH_4_ [13], leading to the enrichment of ^13^CH_4_ and ^12^CH_3_D in the residual methane. A higher combustion efficiency, and possibly higher air-to-fuel ratio, signifies a greater proportion of methane being consumed, resulting in more pronounced isotopic fractionation in the residual methane. The isotopic effect of the combustion process can be simulated with Rayleigh fractionation (gray dashed line in Fig 4):

$${\delta^{13}C}_{residue}(‰)+1000=(1000+{\delta^{13}C}_{initial})*f^{\alpha_{13C/12C}-1}$$

$${\delta D}_{residue}(‰)+1000=(1000+{\delta D}_{initial})*f^{\alpha_{D/H}-1}$$

Here, *f* represents the proportion of residual methane relative to the initial amount of methane prior to combustion. We take the isotopic compositions of the 1999 Volvo S70 cold sample as the initial values. *α* is the kinetic isotope fractionation coefficient, which is the ratio of the rate constant of the heavy isotope to the light isotope in a reaction from the reactant to the product, i.e., k_heavy_/k_light_. We adopt kinetic isotope fractionation factors of ε_13C_ = +7.5‰ (α_13C/12C_ = 0.9926) and ε_D_ = +48‰ (α_D/H_ = 0.9542) as reported by [14]. It should be noted that this set of α values was calculated with the assumption that the observed isotopic variations were attributed solely to the effect of the catalytic converter, while in this study we use them to describe the fractionation attributed to the combustion process. The mechanism would be different, but the approximate magnitude of the Kinetic Isotope Effect (KIE) could be a possible starting point.

Concentration may not show monotonic shifts as isotopes behave, for different exhaust samples that follow Rayleigh fractionation, because it is the proportion rather than the concentration of residual methane that matters. A higher [CH_4_] in the exhaust does not necessarily imply lower combustion efficiency or smaller fractionation. Instead, it could be a consequence of substantial methane production in process #1 methane formation. As shown in Fig 5, process #1 methane formation can generate methane with isotope compositions close to that of gasoline but with varying [CH_4_]. This is followed by process #2 methane combustion, which shifts unburnt methane to more positive δ^13^C values following the gray arrows with fixed slopes proportional to 1/(α_13C, process#1_−1). Process #2 combustion results in the gray area in Fig 5, with the gray dashed line depicting a case in which a higher proportion of methane is combusted if higher [CH_4_] is produced in process #1, thus exhibiting greater fractionation from equilibrium value.

In process #3 catalytic oxidation, unburned methane is expelled into the exhaust system and is further oxidized if a catalytic converter is present. The isotopic effect caused by the catalytic converter might account for the distinct isotope compositions observed in the 1999 Volvo S70 hot sample and two 2017 Chevrolet Sonic hot samples, which have significantly positive δ^13^C and δD values but low [CH_4_]. This process can also be simulated with Rayleigh fractionation, as illustrated by the yellow dash-dotted line in Fig 4, starting with an initial composition of the 1999 Volvo S70 hot sample, coupled with α_13C_ = 0.9963 and α_D_ = 0.9009 to reach the composition of the 2017 Chevrolet Sonic hot when approximately 83% of methane are being oxidized. However, this is an underdetermined system where any values that meet the slope given by (α_D_ -1)/(α_13C_ -1) will fit [63]. When considering concentrations, this Rayleigh fractionation process has a steeper slope proportional to 1/(α_13C, process2_-1), as shown by the yellow arrow in Fig 5, resulting in the yellow area. The greater proportion of methane consumed by the catalytic converter, the lower [CH_4_] and the more positive isotopic composition it becomes. The yellow dash-dotted linear fitting curve does not have physical meaning but suggests that the 1999 Volvo S70 hot and 2017 Chevrolet Sonic hot samples likely have different initial compositions and α values. Figs 4 and 5 simplified the scenario by grouping them under one numerical context.

Diesel vehicles exhibit unique isotope profiles. Diesel engines rely on compression ignition of diesel fuel, which adiabatically heats and ignites the air-rich fuel-poor mixtures, while gasoline engines use spark ignition, which directly ignites the air-fuel mixture with sparks. The higher air-to-fuel ratios in diesel engines result in more complete combustions and notably lower [CH_4_] in the exhaust. One time that diesel engines may operate with more fuel supply would be the trash truck under open throttle acceleration with load (2006 Ford F-750 trash truck hot sample).

Mixing a small amount of fresh air methane into the exhaust could impact the measured isotopic signatures, especially when the [CH_4_] from the uncontaminated exhaust gas is low. This contamination could be the unburnt residue air methane that was sucked into the engine, or fresh air leaked into the sample bags from the outlet of the exhaust pipe during sampling. This mixing effect is supported by the linear fitting trend in Fig 5 (blue dotted line) between the air compositions and two diesel vehicle exhaust compositions, extending to possible compositions of exhaust after significant oxidation by the catalytic converter (yellow area in Fig 5). The isotopic patterns of δ^13^C-δD in Fig 4 also support a mixing trend, with the linear fitting (blue dotted line) pointing to an isotopically highly-enriched endmember. This potential interference of fresh air methane could apply to most of the vehicle samples but should have less impact on higher [CH_4_] samples.

The analysis of isotopic fractionation processes allows us to define several ranges for the isotopic values of methane in vehicle exhaust based on the methane generation process. However, due to limitations in sample size and vehicle composition, these samples inevitably cannot represent the entire US fleet on the road. The methane isotopic signal in vehicle exhaust should not be assigned a single value; instead, it varies across different stages of formation, combustion, and catalytic conversion, ultimately influenced by vehicle operation and maintenance conditions. Generally, exhaust containing moderate concentrations of methane (100 to 1000 ppm) is predominantly influenced by high-temperature methane formation with low combustion consumption, tending to exhibit isotopic signals similar to that of gasoline, with δ^13^C around -21‰ and δD near -170‰. In contrast, high-concentration methane (>1000 ppm) exhaust typically undergoes a higher proportion of methane combustion, resulting in less negative δ^13^C and δD values, where δ^13^C can reach -10‰ and δD approximately -120‰. Furthermore, low-concentration methane (<100 ppm) exhaust, due to the relatively greater contribution of catalytic oxidation effects, tends to have extreme positive isotopic compositions, with δ^13^C possibly reaching 0‰ and δD reaching +100‰.

### Methane clumped isotopologues of vehicle exhaust

**Fig 6:**
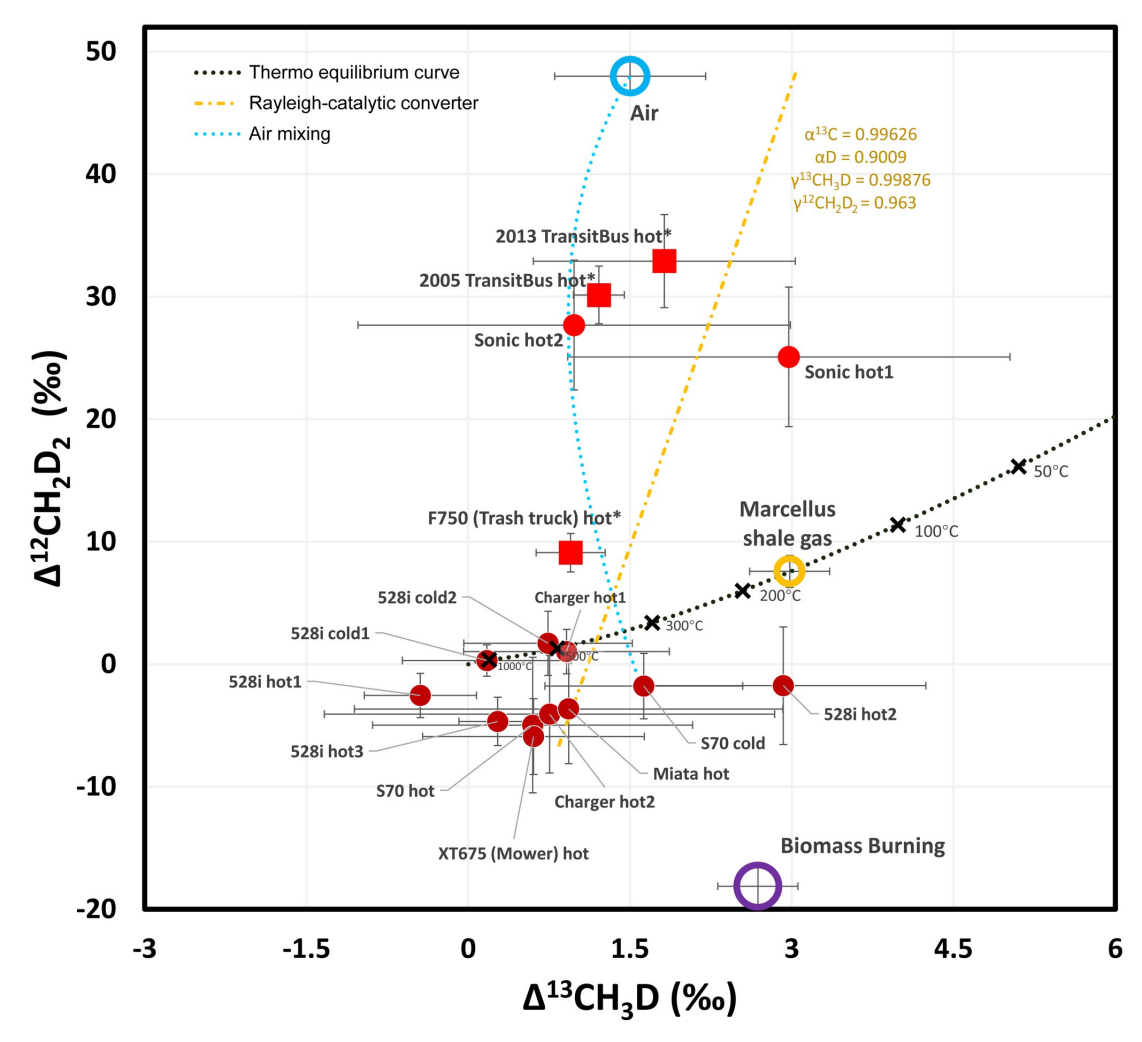
Δ^12^CH_2_D_2_-Δ^13^CH_3_D plot of vehicle exhaust samples. Symbols, curves, and colors are the same as in Fig 4. Error bars are showing 2SD uncertainties after propagation. The black dotted line is the thermo-equilibrium curve for methane clumped isotopologues taken from [64].

The methane clumped isotopologue compositions in most vehicle exhaust samples are slightly anti-clumped in ^12^CH_2_D_2_, and slightly clumped in ^13^CH_3_D (Fig 6 and Table 2). This places vehicle exhaust methane clumped isotopologue compositions close to, but distinct from those of natural gas, and more clumped in ^12^CH_2_D_2_ than biomass burning, pyrolysis [49], and microbial methanogenesis methane clumped signals [35, 39, 40]. Although direct measurements of vehicle exhaust methane yield distinct clumped isotope signals from other methane sources, it is currently not feasible to identify the contribution of vehicle emissions in a road survey by measuring clumped isotopes of methane in ambient air. Unlike nearly pure methane sources such as natural gas leaks and landfill emissions, vehicle exhaust presents concentrations at the ppm level. Incidents where vehicle emissions contribute to a 10% (i.e., ~0.2 ppm) enhancement in methane levels in the environment are quite rare; even when such instances occur, a 10% proportion of vehicle exhaust methane mixed with ambient methane would likely result in the methane isotopic signals being almost completely overwhelmed by the highly positive values of atmospheric methane. Even if we were to observe a slight decrease in Δ^12^CH_2_D_2_, we cannot rule out the possibility that this is due to the mixing of natural gas and/or biogenic methane. Therefore, clumped isotopologues currently cannot be used to distinguish vehicular methane from other methane emissions, and fundamentally, they are not suitable for this task.

Following on the framework proposed in the previous section, which explains the signals of the vehicular methane in four processes–methane formation, combustion, and oxidation via the catalytic converter, and mixing–we can extend this analysis to clumped isotopologues. Methane formation by proton addition to methyl radicals appears to generate anti-clumped compositions like that observed with the burning of wood [34] (biomass burning data here) and pyrolysis of alkanes [49]. According to the experimental results of n-octadecane pyrolysis, the methane production process from alkane pyrolysis could initially generate negative Δ^12^CH_2_D_2_ signals without significantly altering Δ^13^CH_3_D [49]. Once produced, methane will gradually return toward thermal-equilibrium clumped signals as collision and isotopic exchange happens. The low anti-clumping signals we observed imply the dominance of exchanging under high temperatures in the engine.

Process #2 methane combustion and process #3 methane oxidation by the catalytic converter can still be discussed under the framework of the Rayleigh fractionation process, with relationships given by:

$${\delta^{13}CH_{3}D}_{residue}(‰)+1000=(1000+{\delta^{13}CH_{3}D}_{initial})*f^{\alpha_{\frac{13CH3D}{12CH4}}-1}$$

$${\delta^{12}CH_{2}D_{2}}_{residue}(‰)+1000=(1000+{\delta^{12}CH_{2}D_{2}}_{initial})*f^{\alpha_{\frac{12CH2D2}{12CH4}}-1}$$

If we ignore the compounding additional decrease in zero point energy caused by the second rare isotope substitution on a singly-substituted isotopologue (ΔΔZPE, as defined in [45] and similarly described in [65]), according to the rule of the geometric mean [66], the KIE of doubly-substituted isotopologue equals to the product of KIEs of the two corresponding singly-substituted isotopologues, i.e. α_13CH3D_ = α_13C_ * α_D_ and α_12CH2D2_ = α_D_ * α_D_, given that α = 1/KIE. In this scenario, there would be no cap-delta clumping signals, i.e. Δ^13^CH_3_D = 0 and Δ^12^CH_2_D_2_ = 0. Clumping effects are likely small in the case of process #2 methane combustion, where the high-energy environment overwhelms the ΔΔZPE and thus limits clumping.

The presence of a catalytic converter which occurs at lower temperatures in process #3 catalytic oxidation might provide a special surface environment leading to the observed unusual clumping signals in the 2017 Chevrolet Sonic hot exhaust samples. To explain these signals, an additional adjustment coefficient γ for clumping has been introduced, where α_13CH3D_ = α_13C_ * α_D_ * γ_13CH3D_ and α_12CH2D2_ = α_D_ * α_D_ * γ_12CH2D2_. The physical basis for adding this γ coefficient relates to the ΔΔZPE. When Deuterium (D) replaces Hydrogen (H) in a singly-substituted isotopologue, the energy of the existing ^13^C-H or ^12^C-D bond is also affected, which further lowers the total energy of the molecule and makes it more stable, i.e. γ < 1. For ^12^CH_2_D_2_, H-abstraction, which is the main channel for methane oxidation compared to D-abstraction, its secondary isotope effect [45, 67, 68] is largely enhanced because of the existence of two D in the molecule, and is thus expected to cause a stronger clumping effect than ^13^CH_3_D, i.e. usually γ_12CH2D2_ < γ_13CH3D_ < 1. To roughly align the yellow line in Fig 6 with the two 2017 Chevrolet Sonic hot samples, we employed γ_13CH3D_ = 0.99867 and γ_12CH2D2_ = 0.963. The γ_13CH3D_ value deduced in this study aligns numerically with those observed in aerobic oxidation of methane (AOM) experiments [69] and in anaerobic oxidation of methane (AeOM) experiments [50], yet is slightly smaller than the value calculated from the KIEs of gas phase CH_4_+OH reactions [44]. The deduced is γ_12CH2D2_ larger than the measured values in AOM and AeOM samples [42], as well as the calculated value from the KIEs of CH_4_+OH reactions at 25°C [44].

After determining the γ values, KIEs for methane oxidation with the presence of a catalytic converter at high temperature can be calculated: KIE_13CH3D_ = 1.1156 and KIE_12CH2D2_ = 1.2794. These values are significantly lower than those reported for CH_4_+OH reactions at 25°C [44]. Since the catalytic oxidation in vehicle exhaust requires a minimum operational temperature of about 400°C, it is improper to use the 25°C values for comparison. By following established ab initio computational procedures [44] with the Gaussian 09 (EM64L-G09RevD.01) [70] using the second-order Møller-Plesset (MP2) method [71] with cc-pVTZ basis sets [72], we calculated the KIEs for CH_4_+OH and CH_4_+Cl reactions at various temperatures (Table 3), and derived temperature-dependent KIE relationships with fourth-order polynomial fittings (Table 4). We interpolated the KIEs to temperatures of 1283°C and 1116°C, to match the derived KIE_13CH3D_ and KIE_12CH2D2_ in this study, respectively. These temperatures exceed typical exhaust gas temperature when reaching the catalytic converter, which is usually about 600°C.

**Table 3:** Calculated KIEs of four methane isotopologues (^13^CH_4_, ^12^CH_3_D, ^13^CH_3_D, ^12^CH_2_D_2_) for CH_4_+OH and CH_4_+Cl reactions, at different temperatures.

Species		Temprature (K)									
		150	200	253	273	293	298	323	373	500	1000
**OH**	^**13**^ **CH**_**4**_	1.00217	1.00470	1.00602	1.00625	1.00637	1.00639	1.00642	1.00628	1.00541	1.00253
	^**12**^ **CH**_**3**_ **D**	1.31850	1.34051	1.33588	1.33137	1.32598	1.32452	1.31679	1.30000	1.25761	1.15381
	^**13**^ **CH**_**3**_ **D**	1.32347	1.34765	1.34452	1.34022	1.33490	1.33345	1.32565	1.30848	1.26463	1.15679
	^**12**^ **CH**_**2**_ **D**_**2**_	2.02058	2.00236	1.96834	1.94754	1.92421	1.91811	1.88654	1.82188	1.67323	1.35927
**Cl**	^**13**^ **CH**_**4**_	1.02879	1.02473	1.02209	1.02123	1.02042	1.02022	1.01930	1.01767	1.01460	1.00943
	^**12**^ **CH**_**3**_ **D**	1.58934	1.51341	1.46294	1.44489	1.42741	1.42314	1.40246	1.36479	1.29023	1.15734
	^**13**^ **CH**_**3**_ **D**	1.54460	1.47677	1.43110	1.41460	1.39855	1.39463	1.37558	1.34070	1.27120	1.14605
	^**12**^ **CH**_**2**_ **D**_**2**_	2.73024	2.43593	2.25700	2.19441	2.13458	2.12010	2.05073	1.92768	1.69772	1.33611

**Table 4:** Parameters of the derived fourth-order polynomial fittings for calculating KIEs of four methane isotopologues (^13^CH_4_, ^12^CH_3_D, ^13^CH_3_D, ^12^CH_2_D_2_) for CH_4_+OH and CH_4_+Cl reactions with temperatures T in Kelvin. KIE = a / T^4^ + b / T^3^ + c / T^2^ + d / T + e * T + f.

Species		T^-4^	T^-3^	T^-2^	T^-1^	T	1
		a	b	c	d	e	f
**OH**	^**13**^ **CH**_**4**_	-3174600	152620	-1963.8	8.313	1.1173E-06	0.99491
	^**12**^ **CH**_**3**_ **D**	-359920000	7634100	-66793	274.11	0.000035924	0.90329
	^**13**^ **CH**_**3**_ **D**	-350160000	7638700	-68436	283.64	0.000037419	0.89688
	^**12**^ **CH**_**2**_ **D**_**2**_	120570000	5036100	-107210	649.49	0.00010192	0.70991
**Cl**	^**13**^ **CH**_**4**_	1.1703E+07	-113520	-186.91	6.8353	9.8809E-07	1.0019
	^**12**^ **CH**_**3**_ **D**	291190000	-2352300	-12346	196.3	0.000028557	0.94689
	^**13**^ **CH**_**3**_ **D**	254780000	-1878400	-13853	189.78	0.000027486	0.94426
	^**12**^ **CH**_**2**_ **D**_**2**_	1920900000	-24939000	75250	307.09	0.000050941	0.92585

Several approaches could explain the discrepancy in KIE values. One simple explanation is that the KIE for methane catalytic oxidation in vehicle exhaust does have different KIEs than the gas phase CH_4_+OH reaction, similar to how CH_4_+Cl has different KIEs than CH_4_+OH. Another approach might involve adjusting the α_13C_ and α_D_ values defined in the previous section and used in calculating KIEs for clumped isotopologues, which could be both increased while still aligning with measured δ^13^C and δD values, potentially harmonizing the final KIE with those of the CH_4_+OH reaction. Additionally, there could be a method to address the inconsistency without altering the existing values, by considering adsorption-desorption dynamics on the catalytic converter active Pt alloy coating. Methane in the exhaust stroke first adheres to the surface of the catalyst alloy, with part desorbing and another part oxidizing. This would yield a network with two CH_4_ pools and a product:

$$CH_{4}(\mathrm{exhaust})\Leftrightarrow CH_{4}(\mathrm{adsorbed})\Rightarrow \mathrm{Product}$$

At any given moment, assume a fraction *f* of methane in the exhaust stroke is oxidized, and (1-*f*) desorbs. If the methane oxidation on the catalyst surface has KIE, then the net (apparent) KIE’ for the entire network is modulated by the fraction *f*:

KIE′=(1−f)*KIE+f

This equation allows the apparent KIE’ to vary within the range of (1, KIE), controlled by the *f*. The deduced KIE’ values in this study (KIE’_13CH3D_ = 1.1156 and KIE’_12CH2D2_ = 1.2794) can be matched by *f* = 0.36 with interpolated KIE_13CH3D_ = 1.1752 at 600°C and *f* = 0.32 with interpolated KIE_12CH2D2_ = 1.4091 at 600°C for CH_4_+OH reaction, respectively. This consistency in *f* values across different isotopologues strengthens the reliability of using this approach to interpret the clumping effect of the catalytic oxidation process in vehicle exhaust.

If the modified Rayleigh fractionation model incorporating adsorption-desorption processes is adopted, the fractionation of bulk ^13^C and D on the catalytic converter discussed in the previous section should also be explained using the modified model. The previous discussion remains valid as the model still utilizes the mathematical equations of Rayleigh fractionation but instead calculates an apparent fractionation coefficient α’.

Moving to the clumped isotopologue data for diesel vehicles, we find that the trend provided by these data is consistent with the hypothesis of air mixing with the exhaust. The mixing process generates a curve on the Δ^12^CH_2_D_2_-Δ^13^CH_3_D field, depicted by a blue dotted line in Fig 6. The 2006 Ford F-750 trash truck hot sample has a higher contribution from the original exhaust methane, compared to 2005 and 2013 Gillig transit bus hot samples, and thus the isotopic signals were less affected by the air mixing, resulting in a smaller increase in Δ^12^CH_2_D_2_.

To sum up, the observation from the clumped isotopologue data aligns with the conclusions drawn from carbon and hydrogen isotope data. That is, the observed isotopic and isotopologue data can be explained through four processes (formation, combustion, oxidation, and air mixing) combined with appropriate KIE values.

The clumped isotopic values of vehicular methane endmembers should be defined using the same process-based approach as traditional bulk isotopes. However, unlike bulk carbon and hydrogen isotopic data, clumped isotopologue measurements have high uncertainties, while the range of variation is relatively narrow. We observed that fractionation from Step 3 (catalytic conversion) and Step 4 (air mixing) caused the Δ^12^CH_2_D_2_ values of four low-methane concentration samples to diverge significantly from others. Exhaust methane could have Δ^12^CH_2_D_2_ values that become extremely positive, reaching up to +30‰.

Excluding the four outliers, samples clustered near the (0,0) point in Fig 6, which happened to be samples predominantly influenced by Step 1 (formation) and Step 2 (combustion). Averaging these clustered samples yields values of Δ^13^CH_3_D = +0.8‰ and Δ^12^CH_2_D_2_ = -2.4‰. Considering that these samples generally have higher concentrations and may be more representative on the road, we recommend using this set of averaged values as the clumped isotopologue signatures for vehicle exhaust methane endmember.

This set of values is slightly away from the (0,0) point. This minor shift could be attributed to fractionation possibly caused by cracking during the high-temperature methane formation reaction. However, significant measurement uncertainties due to technical limitations, with average 2SD values of ±1.1‰ and ±3.2‰, hinder further quantitative analysis. Given the unique physical significance of the (0,0) in stochastic distribution, and the considerable measurement uncertainties, additional measurements are needed to determine whether the clumped isotopologue signals of vehicle exhaust methane are definitively deviating from (0,0).

To sum up, based on the data from this study, we suggest that the Δ^13^CH_3_D value for vehicle exhaust methane is primarily around 0.8‰ ± 1.1‰, while the Δ^12^CH_2_D_2_ value is approximately -2.4‰ ± 3.2‰, if not being largely affected by catalytic oxidation and air mixing. These values should be used with caution due to their representativeness limitations.

## Conclusions

This study presents findings consistent with prior studies, showing that exhaust from studied vehicles exhibits a broad range of [CH_4_] that vary depending on factors like vehicle age, state of repair, engine type, and operating conditions. [CH_4_] in vehicle exhausts measured in this study ranged from <1 ppm to >1000 ppm. Older and poorly-maintained vehicles tend to emit more methane. Vehicles with compression-combustion (diesel) engines, allowing higher air/fuel, emitted lower [CH_4_] compared to spark-combustion vehicles. Vehicular methane emissions are likely to follow a fat-tail distribution, meaning that a small portion of vehicles contribute the majority of emissions.

The C_2_:C_1_, δ^13^C and δD of exhaust methane vary among vehicles, influenced by many of the same factors that concentrations scale with. C_2_:C_1_ varied from 0.1% to 18.3%. Vehicles emitting greater amounts of methane are generally those with higher C_2_:C_1_. δ^13^C and δD could in principle be used to identify vehicular contributions, but low levels of [CH_4_] enhancements on the road make this a challenging prospect. Vehicular methane typically has δ^13^C values between -22 and -11‰ and δD values between -170 and -120‰, which are less negative than δ^13^C and δD of natural gas. The majority of vehicle samples show a positive linear relationship among [CH_4_], δ^13^C, and δD, although some samples significantly deviate from this trend.

After excluding some outliers, most samples show clumped isotopologue signals clustered near the (0,0) point, with average values of Δ^13^CH_3_D = +0.8 ± 1.1‰ and Δ^12^CH_2_D_2_ = -2.4‰ ± 3.2‰. More high-precision measurements are needed to determine whether these values consistently deviate slightly from stochastic distributions. A small number of outliers can have Δ^12^CH_2_D_2_ values reaching +30‰. The clumped isotopologue signal of vehicle exhaust methane is significantly different from those of major methane sources, such as natural gas and microbial methane.

Despite the isotopic and isotopologue signatures of vehicle exhaust methane being different from other major methane sources, identifying vehicular methane emissions in urban air based on isotopes and isotopologues is still challenging due to the low enhancement level and the variability of isotopic signals from different sources.

The isotopic variations can be explained with four dominant processes: 1) formation of methane under high temperature during the first part of burning in the engine, 2) combustion of methane under high temperature in the engine before it is expelled from cylinder to exhaust stroke, 3) oxidation of methane by catalytic converter, and 4) mixing of exhaust methane with air methane. Process #1 methane formation aligns the δ^13^C and δD values of generated methane with those of gasoline, while clumped isotopologue signals of generated methane are near-stochastic, or exhibit slight anti-clumping in Δ^12^CH_2_D_2_. This process may involve considerable intermolecular exchange of isotopes to yield a near-equilibrium signature. Process #2 methane combustion, described by the Rayleigh fractionation equation, is thought to produce the positive linear relationship observed among [CH_4_], δ^13^C, and δD in the majority of samples. Process #3 methane oxidation by the catalytic converter can also be simulated with a simple Rayleigh model, but a modified Rayleigh model that incorporates methane adsorption and desorption on the surface of the catalytic alloy allows better fit with literature values. The catalytic process leads to extremely positive δ^13^C and δD, as well as highly clumped Δ^12^CH_2_D_2_ signals with low [CH_4_]. The exhaust of diesel vehicles typically shows very low [CH_4_] because compression combustion is generally under a higher air-to-fuel ratio. This low [CH_4_] exhaust is then mixed with ambient air while sampling to yield influenced δ^13^C, δD, and clumped isotopologue signals, which showed mixing trends with air. These processes collectively shape the concentration and isotope characteristics of vehicular methane, indicating vehicle emissions are primarily controlled by the operational states of the engine and catalytic converter. This study demonstrates the capability of isotopes and clumped isotopologues in tracing processes. Meanwhile, vehicle engines and catalytic converters provide a unique opportunity to obtain key isotopic fractionation factors for pyrolysis and methane catalytic oxidation. These parameters could provide a reference for studying other methane geochemical processes, such as microbial methane oxidation and atmospheric methane sink reactions.

## Supporting information

S1 File Additional descriptions and figures of sampling details (Supporting information S1 Note, supporting information S1 Fig), descriptions of sampled vehicles (Supporting information S2 Note), descriptions of concentration measurement results (Supporting information S2 Fig, supporting information S3 Fig), and supplementary discussions of concentration results for some vehicles (Supporting information S3 Note) are provided in the supporting information. (DOCX)
