# Supplementary material for: Controls on concentrations and clumped isotopologues of vehicle exhaust methane
Source: PLoS One. 2025 Feb 21;20(2):e0315304. doi: 10.1371/journal.pone.0315304 (PMC11844870; doi:10.1371/journal.pone.0315304)
Supplement: S1 File — (DOCX) [file pone.0315304.s001.docx]

**Supporting Information** for

Controls on Concentrations and Clumped Isotopologues of Vehicle Exhaust Methane

*Jiayang Sun ^a,b,c *^, Mojhgan A. Haghnegahdar ^a,d^, Julianne M. Fernandez ^a,e^, Cédric Magen ^a^, James Farquhar ^a,f^*

^a^ Department of Geology, University of Maryland, College Park, Maryland 20742, United States

^b^ Air Resources Laboratory, National Oceanic and Atmospheric Administration, College Park, Maryland 20740, United States

^c^ Cooperative Institute for Satellite Earth System Studies (CISESS), University of Maryland, College Park, Maryland 20740, United States

^d^ Smithsonian Environmental Research Center, Edgewater, Maryland 21037, United States

^e^ Global Monitoring Laboratory, National Oceanic and Atmospheric Administration, Boulder, Colorado 80305, United States

^f^ Earth System Science Interdisciplinary Center, University of Maryland, College Park, Maryland 20740, United States

* Correspondence to: [jiayangs@umd.edu](mailto:jiayangs@umd.edu) (J. Sun)

**This file includes:**

Notes S1 to S3 (Page 2-6)

Figures S1 to S3 (Page 7-9)

Note S1. Description of sampling procedures

In the first sampling campaign, methane concentrations in the exhaust were pre-determined before sampling to estimate the required sample volume for isotopic analysis. This involved analyzing methane concentration using a gas chromatograph (GC) of a few milliliters of exhaust gas collected with a syringe. Approximately 800 L of samples are required, if the exhaust is with air-level (2 ppm) methane concentration, to ensure that the amount of collected methane exceeds 70 μmol. Large-volume samplings were achieved using portable pumps to inflate Tedlar bags. The exhaust gas was pulled through a 1-meter metal tube that was partially inserted into the exhaust pipe, then a long (~ 2 m) rubber tube to the pump, and was pushed into bags. In this first sampling campaign, methane and ethane concentrations were measured post-collection. Hence, information on real-time concentration changes was not available.

During the first sampling campaign, we acquired a Mira Ultra LDS Infrared multipass laser gas analyzer (Aeris#302) from Aeris Technologies Inc. (referred to as Aeris hereafter). The acquisition of Aeris enabled us to obtain real-time concentration data for both methane and ethane.

In the second sampling campaign, a 1-meter metal tube was partially inserted into the vehicle’s exhaust pipe, with the other end attached to a drying tube filled with 8-mesh desiccant-anhydrous indicating Drierite and a particle filter before being connected to the Aeris (Figure S1). Participants were instructed to start the engine, initially keep the vehicle in an idling state until a stable concentration signal was recorded. Then they were instructed to rev the engine without load to reach and maintain 3000 rpm for approximately 20 seconds, before returning to an idling state.

Note S2. Description of sampled vehicles

During the first sampling campaign, the 1999 Volvo S70 and 1981 BMW 528i exhaust gases were sampled immediately after a cold start in an idling state, as well as after vehicles warmed up and were revving at 3000 rpm. The rest of the vehicles were sampled after warming up in an idling state. Due to its extremely high concentration levels, the 1981 BMW 528i was sampled again both in cold and hot conditions. The 1970 Dodge Charger and 2017 Chevrolet Sonic exhausts were previously collected during an early validation experiment before the first major sampling campaign. The 2013 Kohler XT675 mower has a small single cylinder 4 stroke engine for domestic use and does not have a catalytic converter. The 1970 Dodge Charger is also not equipped with a catalytic converter due to its age and is one of two carburetor engines. The 1981 BMW 528i was in notably poor condition, exhibiting misfires in some tests, which was diagnosed as a result of a loose plug wire. The 1995 Mazda MX-5 Miata had been stored in the garage for a long term prior to measurement. The Ford F-250 (unknown year) was idling for at least 20 minutes before being sampled. The 2005 and 2013 Gillig transit buses on the UMD campus and a 2006 Ford F-750 trash truck working at University Park, Maryland are diesel-fueled, and equipped with compression-ignition engines which differ from the other vehicles with spark-ignition engines running on gasoline.

In the second sampling campaign, the 2017 Prius Prime Hybrid was operating solely on its battery engine, with no option to switch to the gasoline engine if not moving. The 2013 Nissan Altima expelled some liquid water from the exhaust pipe at the end of acceleration. The 2017 Alfa Romeo Giulia Q4 has a turbocharged engine. The 2009 Honda Accord features a V6 engine, and the 2023 Lexus GX460 is equipped with a V8 engine. Two 2023-model vehicles (2023 Lexus GX460 and 2023 Chevrolet Silverado) were being used for less than a month before joining the test. According to our information, the 2019 Audi A5 had been well-maintained by its owner, however, the 2013 Nissan Altima, 2016 Toyota Camry, and 2018 Toyota RAV4 had poor maintenance histories.

All vehicles not specifically mentioned were gasoline-fueled vehicles with I4 cylinders in normal conditions. We lack data to quantify the conditions of all vehicles.

For multiple samples from the same vehicle under identical conditions, numerical suffixes indicated sampling sequence (e.g., 1970 Dodge Charger 1 for the first sample from the 1970 Dodge Charger, 1970 Dodge Charger 2 for the second). When sampling the same vehicle under different conditions, brief descriptors were used (e.g., 1999 Volvo S70 hot and 1999 Volvo S70 cold). The vehicle conditions were qualitatively described: for the bag samples from the first sampling campaign, "cold" referred to samples taken immediately after the vehicle starts and idles in winter, while "hot" referred to samples taken after at least 10 minutes of driving and then idling. If not labeled "cold" or "hot," the samples were still "hot" but lacked a corresponding "cold" sample. For the second sampling campaign, we measured real-time concentrations during "ignition, idling, revving, and post-acceleration" operational states without distinguishing between "cold" and "hot."

Note S3. Additional discussion of methane emission from each sampled vehicle

The highest methane concentration, exceeding atmospheric levels by a factor of a thousand, was recorded during the cold start and warm up of the 1981 BMW 528i which turned out to have a loose plug wire and misfire. Some vehicles emitted methane at concentrations below ambient levels at certain operation phase, such as the 2023 Chevrolet Silverado with a 2.7L Turbo I4 engine and the 2019 Hyundai Kona while idling. The 1981 BMW 528i provides an example of a vehicle emitting due to age and poor maintenance. In this case, a loose plug wire resulted in an engine misfire allowed unburnt fuel to bypass the combustion process and be routed directly to the catalytic converter. Under normal conditions, the catalytic converter facilitates the conversion of unburned hydrocarbons to CO_2_. In the presence of substantial unburned gasoline, catalytic converter still operates, but the process can instead lead to incomplete conversion of gasoline into CO_2_ and instead produce lower hydrocarbons, such as ethane and methane, thereby functioning as a methane generator, and this was the case with this 1981 BMW 528i. The 2013 Kohler XT675 mower and 1970 Dodge Charger, both lack catalytic converters, demonstrated exhaust methane concentrations representative of post-combustion gases under their respective operating conditions. Based on our familiarity with poor maintenance histories of the 2013 Nissan Altima, 2016 Toyota Camry, and 2018 Toyota RAV4, we surmise that inadequate maintenance contributed to elevated emissions. In contrast, the 2023 Chevrolet Silverado and 2023 Lexus GX460, both in pristine condition, have the lowest emissions. The 2019 Audi A5 and 2019 Hyundai Kona were known for having good maintenance, also showed low methane emissions. Notably, some vehicles manufactured between 2015 and 2018, which make up a significant part of current urban fleets, have high emission levels. This suggests that vehicle’s maintenance status, rather than its manufacturing year, plays a more crucial role in determining emission levels.

Diesel vehicles generally emit exhaust with low methane concentrations, potentially because they are equipped with compression-ignition engines, which have higher air-to-fuel ratios compared to spark-ignition gasoline engines, thereby achieving enhanced combustion efficiency. However, heavy-duty diesel engines, such as the 2006 Ford F-750 trash truck that frequently operate under loads, have elevated emissions.

However, this dataset should not be used to quantitatively estimate the average exhaust methane concentration, given that our sampling design was biased on purpose and was constrained to accessible vehicles.


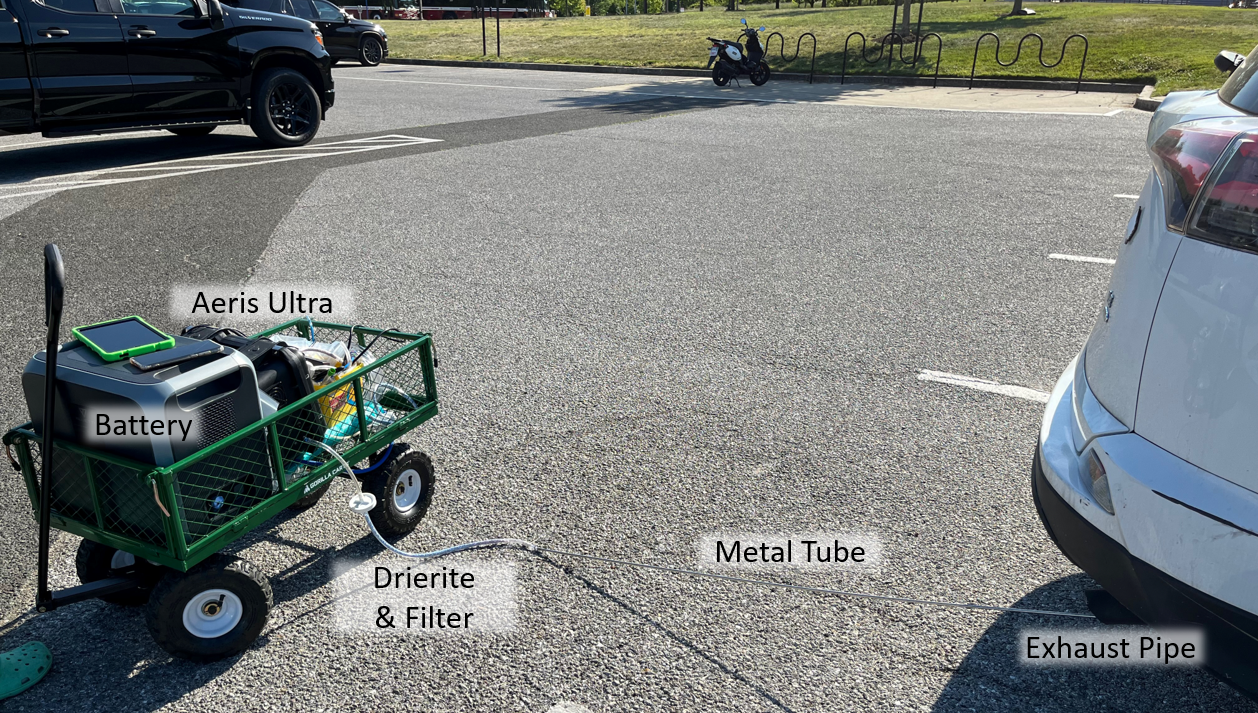


Figure S1. Photo while monitoring the real-time methane and ethane emissions from the 2018 Toyota RAV4 using Aeris, during the second sampling campaign.


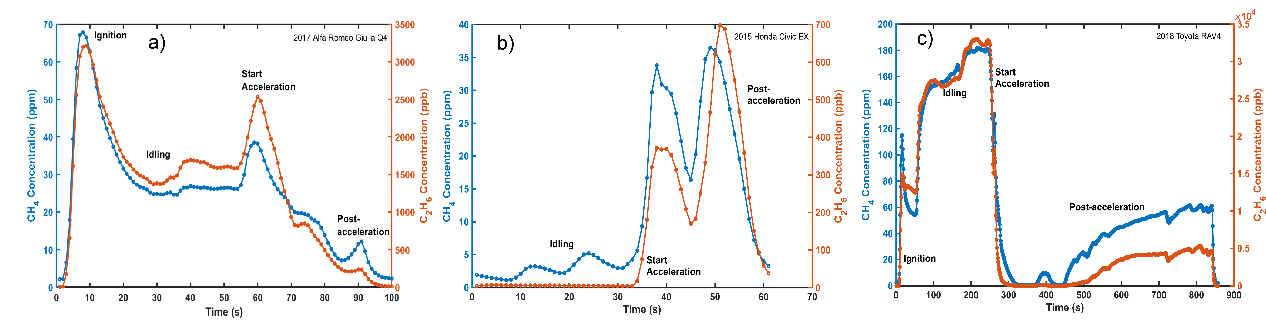


Figure S2. Continuous monitoring of a) 2017 Alfa Romeo Giulia Q4, b) 2015 Honda Civic EX, c) 2018 Toyota RAV4 exhaust methane and ethane concentration throughout operation cycles (ignition, idling, revving, and post-revving).


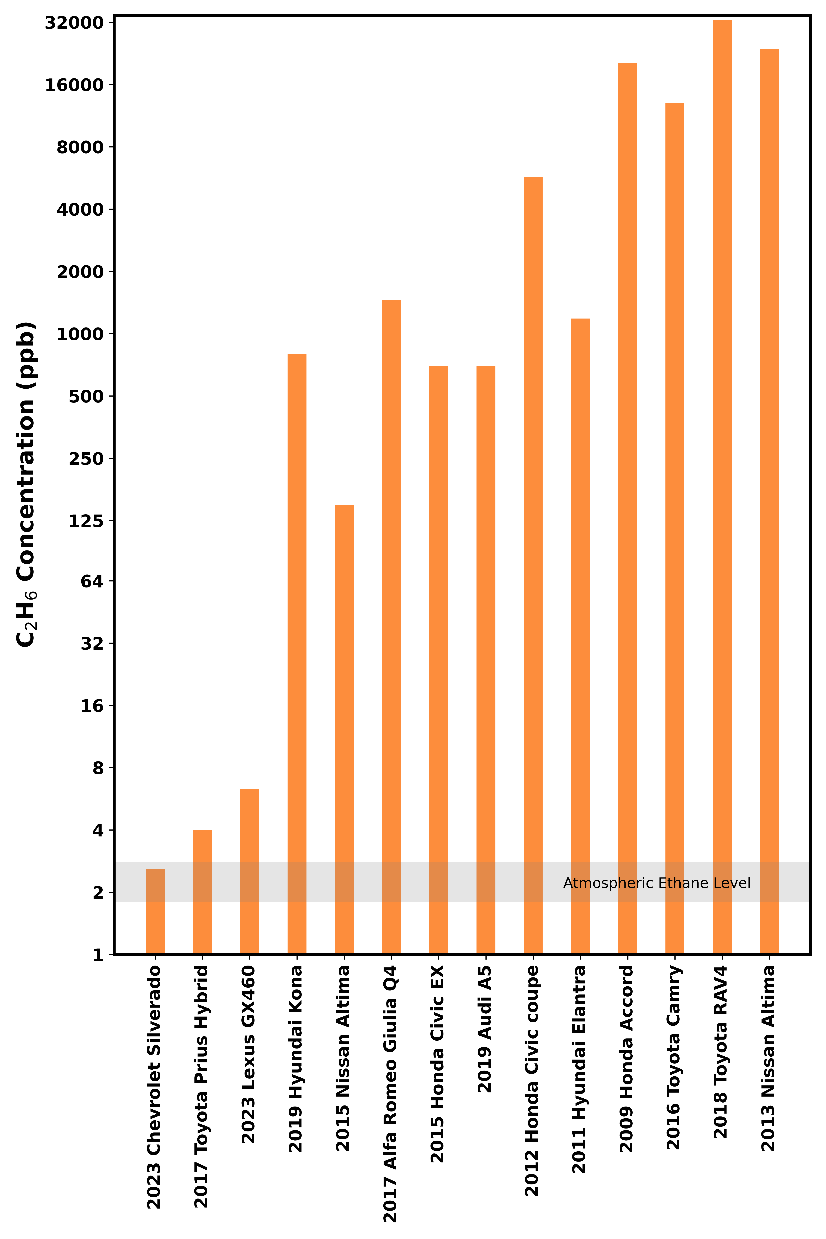


Figure S3. Ethane concentrations of vehicle exhaust samples. The sequence on X-axis is basically consistent with the sequence in Figure 2, except that the vehicles in the first campaign have no ethane information and are not included. The plotted concentrations are peak values among the vehicle operational cycles; therefore, the heights of the bars are not fully representative of the total emission fluxes from the corresponding vehicles. Asterisks (*) in labels mark diesel vehicles. The Y-axis is scaled logarithmically. Detailed information on individual vehicles can be found in Section 2.1, Table 1, and Supporting Information Note S2.
